# The newest TRP channelopathy: Gain of function TRPM3 mutations cause epilepsy and intellectual disability

**DOI:** 10.1080/19336950.2021.1908781

**Published:** 2021-04-14

**Authors:** Siyuan Zhao, Tibor Rohacs

**Affiliations:** Department of Pharmacology, Physiology and Neuroscience, Rutgers, New Jersey Medical School, Newark, NJ, USA

**Keywords:** TRPM3, Channelopathy, Epilepsy, TRP channel

## Abstract

Transient Receptor Potential Melastatin 3 (TRPM3) is a Ca^2+^ permeable nonselective cation channel, activated by heat and chemical agonists, such as the endogenous neuro-steroid Pregnenolone Sulfate (PregS) and the chemical compound CIM0216. TRPM3 is expressed in peripheral sensory neurons of the dorsal root ganglia (DRG), and its role in noxious heat sensation in mice is well established. TRPM3 is also expressed in a number of other tissues, including the brain, but its role there has been largely unexplored. Recent reports showed that two mutations in TRPM3 are associated with a developmental and epileptic encephalopathy, pointing to an important role of TRPM3 in the human brain. Subsequent reports found that the two disease-associated mutations increased basal channel activity, and sensitivity of the channel to activation by heat and chemical agonists. This review will discuss these mutations in the context of human diseases caused by mutations in other TRP channels, and in the context of the biophysical properties and physiological functions of TRPM3.

## Transient receptor potential (TRP) channels

The mammalian TRP superfamily has more than 20 members. TRP channels are activated by a variety of chemical and physical stimuli. They have six transmembrane domains and long cytosolic N- and C-termini, and they are distantly related to voltage-gated Na^+^, K^+^ and Ca^2+^ channels [1]. Functional TRP channels are homotetramers, and in some cases heterotetramers. Most TRP channels are outwardly rectifying Ca^2+^ permeable cation channels, even though there are exceptions to this general behavior. Based on the sequence homology and structural similarity, mammalian TRP channels are divided into six subfamilies: the classic TRPC, the vanilloid TRPV, the melastatin TRPM, the ankyrin TRPA, the polycystin TRPP, and the mucolipin TRPML [2]. TRP channels are involved in many different physiological processes, including temperature sensing, taste, vision, nociception, epithelial ion transport, and mineral homeostasis.

## Mutations in human TRP channels cause channelopathies

Channelopathy means ion channel disease, which can arise in a number of different ways [3], but the term is most commonly used to denote diseases that are caused by mutations in ion channel genes. Mutations in TRP channels cause several different diseases [4]. Table 1 shows a summary of currently known human diseases that are caused by, or associated with TRP channel mutations. Consistent with the involvement of TRP channels in a wide variety of physiological functions, their mutations affect a number of different organ systems.

**Table 1.** Human diseases caused by mutations in TRP channels

Channel	Functional effect	Disease	Reference:
TRPC6	Gain of function	Focal Segmental Glomerulosclerosis	[18]
TRPV3	Gain of function	Olmsted Syndrome (skin disorder)	[20]
TRPV4	Gain of function	Brachyolmia type 3	[27]
TRPV4	Gain of function	Spondylometaphyseal dysplasia, metatropic dysplasia	[28]
TRPV4	Gain of function	Congenital distal spinal muscular atrophy	[29]
TRPV4	Gain of function	Scapuloperoneal hereditary motor neuropathy	[30]
TRPV4	Loss of function	Hyponatremia	[31,33]
TRPV5	Loss of function	Kidney stones	[16,17]
TRPV6	Loss of function	Transient neonatal hyperparathyroidism (TNHP);	[12]
TRPV6	Loss of function	Early-Onset Chronic Pancreatitis	[14]
TRPV6	Gain of function	Kidney stones	[15]
TRPM1	Loss of function	Congenital Stationary Night Blindness	[6–8]
TRPM2	Loss of function	Western Pacific Amyotrophic Lateral Sclerosis (ALS) and Parkinsonism Dementia (PD)	[26]
TRPM3	Gain of function	Intellectual disability and epilepsy	[35–37,71]
TRPM4	Gain of function	Progressive familial heart block type I (PFHBI)	[22]
TRPM4	Gain of function	Progressive Symmetric Erythrokeratodermia	[21]
TRPM6	Loss of function	Hypomagnesemia with secondary hypocalcemia	[10,11]
TRPM7	Loss of function	Western Pacific Amyotrophic Lateral Sclerosis (ALS) and Parkinsonism Dementia (PD)	[25]
TRPA1	Gain of function	Familial Episodic Pain Syndrome	[5]
TRPML1	Loss of function	Mucolipidosis type IV	[23,24]
TRPP2	Loss of function	Autosomal dominant polycystic kidney disease (ADPKD)	[19]

In agreement with the well-established roles of TRP channels in somatosensation and nociception in animal models, gain of function mutations in the mustard oil sensitive noxious chemical sensor TRPA1 channels lead to a spontaneous pain syndrome [5]. Despite the enormous attention TRP channels received in the pain field, TRPA1 is the only known TRP channel so far mutation of which causes a pain related phenotype in humans. TRP channels also play roles in sensory functions other than somatosensation. Loss of function mutations in TRPM1 cause Congenital Stationary Night Blindness in humans [6–8]. Interestingly, mutations in TRPM1 are also associated with stationary night blindness and depigmentation in Appaloosa horses [9].

Some TRP channels play key roles in epithelial ion transport. TRPM6 for example is an epithelial Mg^2+^ transport channel in the kidneys and in the intestines, and loss of function mutations in this channel cause hypomagnesemia [10,11]. TRPV6 is an epithelial Ca^2+^ transporting channel. It is expressed in a number of different organs, including the intestines and the placenta, and it is involved in organism level Ca^2+^ homeostasis. Consistent with this role, loss of function mutations in TRPV6 cause Transient neonatal hyperparathyroidism (TNHP) [12], or severe undermineralization and dysplasia of the fetal skeleton [13]. Loss of function mutations of TRPV6 were also found in patients with early onset non-alcohol related pancreatitis [14]. A mild gain of function ancestral variant of TRPV6 was associated with kidney stones, presumably because increased Ca^2+^ absorption in the intestines through TRPV6 was compensated by increased excretion of Ca^2+^ in the urine [15]. Kidney stone risk is also associated with loss of function mutations in TRPV5, a Ca^2+^ transporting channel expressed in the kidney [16,17].

Gain of function mutations in TRPC6 channels cause Focal Segmental Glomerulosclerosis, likely mediated by Ca^2+^ overload of podocytes and as a consequence a damage to the filtration barrier leading to decreased kidney function [18]. Loss of function mutations in TRPP2, also known as polycystin 2, lead to Autosomal dominant polycystic kidney disease [19].

TRP channels are also found in the skin. TRPV3 is expressed in keratinocytes, where it is activated by warm temperatures. Its gain of function mutations in humans cause Olmsted Syndrome, a rare and severe skin disorder characterized by palmoplantar and periorificial keratoderma, alopecia and itch [20]. TRPM4 is Ca^2+^ impermeable nonselective cation channel, activated by elevated cytoplasmic Ca^2+^. It is expressed in a variety of tissues including keratinocytes. A recent report showed that gain of function mutations in this channel cause Progressive Symmetric Erythrokeratodermia, a skin disease characterized by sharply demarcated hyperkeratotic, erythematous plaques. TRPM4 mutants described in this disorder showed substantial baseline activity, and enhanced sensitivity to intracellular Ca^2+^, but no increase in cell surface protein levels [21]. It is noteworthy that different TRPM4 mutations described earlier cause Progressive familial heart block type I. Those mutations lead to gain of function via attenuated desumoylation leading to impaired endocytosis and increased channel density at the cell surface [22].

Mutations in TRP channels may also lead to neurodegenerative diseases. TRPML1 is an endolysosomal channel, that gave name to the mucolipin family. Mutations in this channel cause mucolipidosis type IV, an autosomal recessive developmental neurodegenerative disorder characterized by severe psychomotor retardation and ophthalmologic abnormalities. The pathomechanism of this lysosomal storage disease is likely to be a defect in the late steps of endocytosis [23,24]. Loss of function mutations in TRPM7 [25] and TRPM2 [26] have been associated with Western Pacific Amyotrophic Lateral Sclerosis (ALS) and Parkinsonism Dementia (PD).

When it comes to TRP channelopathies, TRPV4 is in its own league. Gain of function mutations in this channel are associated with a dizzying array of somewhat overlapping osteoarticular disorders and neuropathies. These disorders include Brachyolmia type 3 [27], Spondylometaphyseal dysplasia (Kozlowski type), metatropic dysplasia [28], congenital distal spinal muscular atrophy [29], and Scapuloperoneal hereditary motor neuropathy [30]. TRPV4 is a well-established osmosensitive channel [31], also activated by heat, thus this disease pattern came as a surprise, and the pathomechanism of how mutations of this channel lead to such a diverse set of abnormalities is not clear [32]. Interestingly, but consistently with its osmosensor role, loss of function mutations in TRPV4 are associated with hyponatremia [33].

TRPM3 is a heat-activated ion channel, and its role in noxious heat sensation in mice is very well established [34]. Thus, it came as somewhat of a surprise that a recent publication identified mutations in this channel in patients with developmental and epileptic encephalopathy, a disorder characterized by seizures and intellectual disability [35]. Subsequent publications found that the disease-associated mutations resulted in overactive TRPM3 channels, with increased basal activity, and increased heat and agonist sensitivity [36,37]. The remainder of this review will discuss the functional effects of these mutations as well as potential pathomechanism of this disease in the context of the known properties of TRPM3.

## TRPM3 channels

The human TRPM3 gene is located on chromosome 9 (9q21.11-q21.12) and it contains 28 exons and several huge introns, including the largest one on this chromosome [38]. Because of different transcription starts sites, TRPM3 may present as TRPM3α variants, which start with exon 1 and lacks of exon 2 [39], and TRPM3β variants, which start with exon 2 [40]. Variants of human TRPM3 starting with exon 4 have also been described [41]. Several other exons also undergo alternative splicing, and as a result TRPM3 has a large number of splice variants [40]. Even though the functional relevance of many of the splice variants remains incompletely understood, functional differences between some of the variants have been identified. For example, the TRPM3α1 splice variant was reported to have lower divalent cation permeability compared to the most widely used and characterized TRPM3α2 [39].

Similar to most TRP channels, TRPM3 is a calcium permeable, nonselective cation channel with six transmembrane domains and a pore region formed between transmembrane segments S5 and S6 [42]. TRPM3 is activated by the endogenous neurosteroid Pregnenolone Sulfate (PregS) and by the synthetic agonist CIM0216 [41]. TRPM3 channels are also activated by high temperatures. As one of the common features of TRP channels, the activation of TRPM3 requires the presence of phosphatidylinositol 4,5-bisphosphate (PIP_2_) [43,44]. Unlike other TRP channels, TRPM3 was reported to have an alternative permeation pathway in S4, which is also known as alternative pore [45]. Distinct from the central pore, the alternative pore shows inwardly rectifying characteristics and it can be activated by the combined application of PregS and clotrimazole [45] or CIM0216 alone [46].

TRPM3 is expressed in peripheral sensory neurons of the dorsal ganglia (DRG) both in mice [47] and in humans [48]. Consistent with heat activation of these channels, TRPM3 deficient mice showed reduced ability to detect noxious heat [47], and TRPM3-TRPV1-TRPA1 triple knockout mice completely lost their ability to detect high temperatures [49]. TRPM3 also plays a role in heat hypersensitivity in inflammatory [50] and neuropathic pain [51] in mice. TRPM3 is also expressed in pancreatic β-cells where it may play a role in regulating insulin release [52]. TRPM3 is also expressed in the spinal cord, and various regions of the brain, including the hippocampus, locus coeruleus, cerebellum and hypothalamus [40], but relatively little is known about the functional role of TRPM3 in the central nervous system. The channel was shown to be present both in neurons [53,54] and in oligodendrocytes [53]. In cerebellar Purkinje neurons, TRPM3 activation was shown to increase glutamatergic transmission [54]. In vagal afferents in the nucleus of the solitary tract TRPM3 was shown to control both basal and temperature-driven glutamate release [55].

Several different inhibitors of TRPM3 have been described. Flavonones, such as isosakuranetin and liquiritigenin, were shown to inhibit TRPM3 with nanomolar EC_50_. Consistent with the role of TRPM3 in thermal nociception, isosakuranetin increased the latency of nocifensive responses on a hot plate in mice [56]. Primidone, a clinically used antiepileptic drug was also shown to inhibit TRPM3 channel activity, and thermal nociception in mice [57]. Primidone is a pro-drug; its antiepileptic activity is thought to be due to its conversion to anticonvulsant barbiturates in the liver. Whether the more recently described direct inhibitory effect of primidone on TRPM3 contributes to its antiepileptic effects is not known.

TRPM3 activity is also tightly regulated by G-protein coupled receptors (GPCR-s). In DRG neurons, activation of Gαi-coupled receptors such as opioid or GABA_B_ receptors robustly inhibits TRPM3 activity through the binding of Gβγ subunits to the channel [58–60]. It was recently reported that Gβγ inhibits TRPM3 by binding to a 10 amino acid segment in TRPM3, encoded by an alternatively spliced exon that is missing in some naturally occurring splice variants [61]. TRPM3 activity can also be inhibited by Gq-coupled cell surface receptors also mainly via Gβγ [59,62].

## Gain of function mutations in TRPM3 cause epilepsy and intellectual disability

As one of the most common neurologic disorders, epilepsy affects approximately 50 million people worldwide [63]. It can occur at different ages with many possible clinical syndromes and causes such as genetic mutations and malformations of cortical development [64,65]. In epilepsy, normal electrical activity in the brain is disrupted by sudden, synchronized bursts, resulting in recurrent seizures. Mutations in ion channels can cause epilepsy [66]. Generally, either a loss of function of K^+^ channels, or a gain of function of Na^+^ or nonselective cation channels increases excitability of neurons and thus can lead to epilepsy. For example, *de novo* dominant negative mutations in voltage-gated potassium channel subfamily C member 1 (KCNC1) were identified in epilepsy patients [67]. Gain of function mutations in various voltage-gated Na^+^ channels leading to increased persistent current were also found to cause epilepsy [68]. Developmental and epileptic encephalopathies (DEE) are characterized by epilepsy and comorbid intellectual disability. Mutations in ion channels were also identified as causes for DEE [69]. Moderate loss of function mutations in the KCNQ2 channel was among the first ion channel mutations identified that could cause DEE [70].

**Figure 1.**
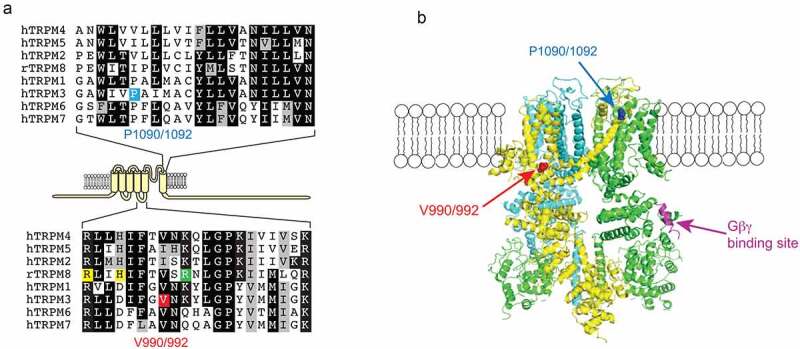
Location of the disease-associated TRPM3 mutant residues

A recent publication identified recurrent *de novo* mutations in TRPM3 channels in pediatric patients with DEE [35]. They found eight unrelated probands who showed moderate-to-severe developmental delay and epilepsy. Some of the patients also showed other symptoms, such as scoliosis (three out eight patients) and strabismus (crossed eyes, four out of eight patients). In the following genetic investigation, they found that seven patients were heterozygous for the recurrent *de novo* TRPM3 mutation V837M (V990M), which is located in the S4-S5 linker (Figure 1). Note that the numbering of these residues differs between different splice variants of TRPM3, see Figure 1 legend for details. Another patient showed heterozygous expression of the specific TRPM3 mutation P937Q (P1090Q) which is located in the outer pore region of the channel (Figure 1). However, the functional effects of these two mutations on channel activity were not tested.

A: Top panel: Sequence alignment of the outer part of S6. The P1090/1092 residue in TRPM3 is highlighted blue. P1090 is the location of this residue in splice variant hTRPM3_1325_ used by both Zhao et al. [37] and Van Hoeymissen et al. [36] for functional studies, P1092 is the same reside in the hTRPM3α2 splice variant also used for functional studies by Zhao et al. [37]; they correspond to P937 in Dyment et al. [35]. Bottom panel: Sequence alignment of the S4-S5 loop, the V990/992 residue in TRPM3 is labeled red, V990 and V992 are the locations of the same residue in hTRPM3_1325_ and hTRPM3α2, and they both correspond to V837 in Dyment et al. [35].Yellow highlights indicate residues in contact with the agonists icilin and/or WS12 in the TRPM8 structures (6nr3, 6nr4, 6nr2), the residue highlighted green is in contact with PI[4,5]P_2_ in TRPM8. B: the location of residues equivalent to V990/992 and P1090/1092 on the structure of TRPM4 (6bwi); one subunit is hidden for visibility. The short α-helical segment equivalent to the Gβγ binding site in TRPM3 is also shown. Adapted from Zhao et al. 2020 [37].

In a subsequent publication, another research group reported a single case of 5-year-old girl with the same V837M (V990M) mutation, but different phenotypes. Even though the patient presented neurodevelopmental delay, intellectual disability (ID), hypotonia and similar facial characters to the ones earlier reported, she did not have epilepsy [71]. However, it cannot be ruled out that the patient is still young and epilepsy will develop later. This patient was also intolerant to heat, and had a diminished pain threshold, which is consistent with the role of TRPM3 as a heat sensor in nociceptive neurons. Additionally, the patient showed amblyopia (“lazy eye”), impaired vision in one eye due to the brain failing to process inputs from the other eye. This symptom, together with strabismus reported in the first study [35], links TRPM3 with ocular defects, which have been reported earlier both in mice and in humans. TRPM3^−/-^ mice showed deficits in pupillary light responses both in bright and dim light conditions; this defect however was less severe than that observed in TRPM1^−/-^ mice [72]. In inherited cataract in humans, a heterozygous A-to-G transition in exon 3 of TRPM3 was detected. This transition would lead to alternative splicing of TRPM3 and cause codon 65 mutated from isoleucine to methionine (I65M) [73]. Recently it was shown the introduction of the I65M mutation in a knock in mouse line leads to the development of cataracts [74]. Interestingly, the functional effect of this mutation on TRPM3 channel activity was not tested.

## Epilepsy-related TRPM3 mutants have higher basal activity

The hallmark of epilepsy is recurrent seizures which is related to the hyperexcitability of a group of neurons. TRPM3 is a Ca^2+^ permeable nonselective cation channel, opening of which results in Na^+^ influx, which depolarizes the cell, and Ca^2+^ influx, which increases cytoplasmic Ca^2+^ and also contributes to depolarization. Both depolarization and increased cytoplasmic Ca^2+^ lead to increased excitability. Therefore, the first assumption of why the mutated TRPM3 could induce epilepsy would be that mutated TRPM3 channels have increased basal activity.

Following the identification of the two disease-associated TRPM3 mutations V990M and P1090Q, two groups examined their effects on intracellular calcium levels [36,37]. Both these recent publications found that cells expressing either of the mutants, had higher basal calcium levels than cells transfected with wild type TRPM3 at room temperature, and cells expressing the V990M mutant had higher basal Ca^2+^ levels than those expressing P1090Q [36,37]. Additionally, application of TRPM3 inhibitors, primidone [37] or isosakuranetin [36] significantly reduced basal intracellular calcium levels in cells expressing either of the mutant channels, but not in cells expressing the wild type TRPM3. Neither primidone, nor isosakuranetin brought the calcium levels of mutants back to the same level as in the wild type group, see following section for further details. Electrophysiology experiments with channels expressed in HEK293 cells [36] and in Xenopus oocytes [37] showed similar conclusions, basal current activity showing the following pattern of amplitudes: V990M > P1090Q > wild type TRPM3.

The higher basal activities of the mutants likely cause depolarization and elevated Ca^2+^ levels in neurons expressing these channels, which provides a mechanism to induce increased excitability and seizures. The experiments described here were performed at room temperature. Given the heat sensitivity of TRPM3, basal activity of the mutants at body temperature *in vivo* is likely to be even higher, see discussion on temperature sensitivity later.

## Epilepsy-related TRPM3 mutants are more sensitive to chemical agonists and less sensitive to antagonists

**Figure 2.**
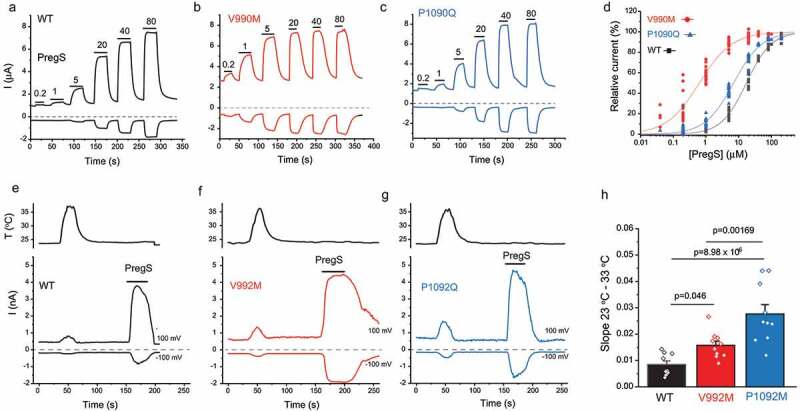
The effect of disease-associated TRPM3 mutations on agonist sensitivity and heat sensitivity

The most widely used TRPM3 agonist is PregS. Both the V990M and P1090Q showed increased sensitivity to PregS compared with wild type TRPM3 in cytoplasmic Ca^2+^ measurements in HEK293 cells [36,37], and in current measurements in Xenopus oocytes [36]. In both publications, the left shift in the concentration response curves for V990M was more pronounced than for the P1090Q mutant [36,37], see also Figure 2A-D, redrawn from Zhao et al. [37].

CIM0216 is also wildely used as a TRPM3 activator. An important difference between PregS and CIM0216 is that CIM0216 also opens the alternative pore of TRPM3 [46]. In cytoplasmic Ca^2+^ measurements both mutants showed higher sensitivity to CIM0216 activation than wild type TRPM3 [37]. Similar to PregS activation, the V990M mutant had a larger left shift than the P1090Q mutant. In conclusion, both disease-related mutations made the channel more sensitive toward PregS and CIM activation, but the V990M mutation produced a larger effect for both agonists than the P1090Q mutation, similar to their effects on basal activity.

Both groups also tested sensitivity to inhibition by the TRPM3 antagonist primidone. The two studies agreed that PregS-induced Ca^2+^ signals were the least sensitive to inhibition by primidone in cells expressing the V990M mutant, followed by the P1090Q mutant, and wild type channels were the most sensitive [36,37]. The V990M mutant was also less sensitive to Ca^2+^ induced inactivation than wild type or P1090Q [36].

Overall, detailed testing of sensitivity of mutant and wild type channels to pharmacological activators and inhibitors by two different labs gave a highly consistent picture. V990M shows the highest basal activity, the highest sensitivity to agonists, and it is the least sensitive to inhibition by antagonists. The P1090Q mutant was in the middle in all assays, and the wild type TRPM3 showed the lowest basal activity, the lowest sensitivity to agonists, and the highest sensitivity to antagonist inhibition.

## Epilepsy-related TRPM3 mutants are more sensitive to temperature activation

TRPM3 is activated at high temperatures, and the temperature dependence of TRPM3 is such that it shows activity at body temperature [47]. Consistent with this, when the temperature was increased from room temperature to 37°C, both wild type TRPM3 and the mutants showed increased intracellular calcium levels. The increases in the mutated TRPM3 channels were two-three times higher than in the wild type group, with P1090Q showing the largest increase, when normalized to the responses induced by maximal agonist (PregS) stimulation [37]. Additionally, when the transfected HEK cells were stimulated with a temperature ramp from 24°C to 40°C, both mutants showed larger calcium influx compared with wild type and the P1090Q showed the largest relative response among the three groups [36].

Since calcium is an indirect indicator of TRPM3 activity, whole cell patch experiments were also performed to test temperature sensitivity of the mutants [37]. The slope of the increase in currents induced by a temperature ramp from 23°C to 36°C as a function of temperature was significantly steeper for both mutants than the wild type group, see Figure 2e-h, redrawn from Zhao et al. [37]. Consistent with the Ca^2+^ imaging data, the P1090Q mutant showed a significantly steeper slope upon temperature activation than V990M (Figure 2h). The data from Ca^2+^ measurements and whole cell patch clamp experiments indicated that both disease-associated mutants had higher sensitivity to temperature activation than wild type TRPM3, and P1090Q had higher sensitivity than the V990M mutant, even though the latter showed a larger effect on both basal activity, and agonist sensitivity.

## Epilepsy-related mutations make TRPM3 overactive via different mechanisms

The data discussed so far show that the V990M mutation had a more robust effect on basal activity and agonist sensitivity, whereas the P1090Q mutation had a larger effect on temperature sensitivity. Do these mutations affect channel activity via different mechanisms? Given the well-known allosteric interactions between temperature and chemical agonists in thermosensitive TRP channels [75], increased temperature sensitivity is expected to also lead to increased agonist sensitivity and vice versa. The most straightforward interpretation of the data is that the V990M mutation primarily affected agonist sensitivity and/or open state stability, which caused a smaller secondary increase in temperature activation. Conversely, the P1090Q mutation may primarily affect temperature sensitivity, which leads to a smaller secondary effect on basal activity and agonist sensitivity. Based on channel topology and cryoEM structures of related TRPM channels (Figure 1) the two residues are in very different locations, and structural and functional data on related TRP channels are compatible with such model, as discussed below.

Adapted from Zhao et al. 2020 [37]. A-C: Representative two electrode voltage clamp experiments in Xenopus oocytes expressing the hTRPM3_1325_ splice variant (A) and its mutants V990M (B) and P1090Q (C). Horizontal lines show the applications of different concentrations of PregS (μM). D: Hill fits of the concentration dependence of the effect of PregS. E-G: Representative whole cell patch clamp measurements on the human TRPM3α2 (A) or its mutants V992M (B) and P1092Q (C) expressed in HEK293 cells. Note that the numbering of the residues differs by 2 because of the different splice variant used compared to (A-C). The applications of 100 μM PregS are indicated by the horizontal lines, the temperature curves on the top panels. **D**: Summary of the slopes of the current increases between 23°C and 33°C. See Zhao et al. 2020 [37] for more details.

The V990M is located in the S4-S5 linker, which has been shown to play essential roles in gating of many TRP channels, including channels from different subfamilies such as TRPA1 and TRPV4 [76]. Accordingly, this segment is a hotspot for gain of function and some loss of function mutations, including naturally occurring disease causing ones in a variety of TRP channels [76]. The S4-S5 linker also plays important roles in ligand binding for many TRPM channels [77]. In the CryoEM structure of TRPM8 channels, the S4-S5 linker has direct contact with its agonists WS12 and the cooling agent icilin [78]. There is no available structure for TRPM3, but given the similarities between TRPM3 and other TRP channels, it is possible that this segment also plays a role in agonist binding to TRPM3. Since PregS is thought to activate TRPM3 through direct binding [79], it is possible that the V990M mutation changes the binding properties of TRPM3 agonists. The activity of TRPM3 also requires the presence of PIP_2_. As the co-structure of TRPM8 with PIP_2_ shows the involvement of the S4-S5 segment in lipid binding, the V990M mutation may also influence the channel activity indirectly through PIP_2_.

What is the mechanism by which the V990M mutation causes the observed increase in basal activity and agonist sensitivity? Given the increased basal activity in the absence of exogenous agonist, a pure increase binding affinity for PregS and CIM0216 can be safely excluded. An increase of the open state stability of the channel on the other hand would explain both the increased basal activity, the increased agonist sensitivity and the decreased sensitivity to antagonists [80]. As PIP_2_ is always present in the plasma membrane, increasing its affinity for the channel may also cause increased basal activity. Given the allosteric interaction between exogenous ligands and PIP_2_ in other TRP channels, increasing PIP_2_ affinity may also indirectly increase apparent affinity for exogenous ligands [81].

The P1090 residue is located in the outer region of S6 and close to the pore loop. Mutation of this residue had a larger effect on the activation by temperature. Even though the mechanisms of heat activation of TRPM3 is not known, experiments on other thermo-TRP channels indicated the potential roles of S6. For instance, the outer pore region of TRPV1 has been proposed to act as a heat sensor [82]. Also, in the two-step activation of TRPV3 by heat, the first highly temperature-dependent step relies on the S6 helices. During the activation, the S6 undergoes α-to-π transition and helps the pore open [83]. Therefore, the possible mechanism of why P1090Q makes the channel more sensitive to temperature activation could be that the mutation influences the temperature-dependent gating properties of TRPM3.

In addition to the differential effects of the two mutations on heat and agonist sensitivity, there were also differences between the two mutations in how they affect the opening of the alternative pore of TRPM3. As mentioned earlier, Clotrimazole alone does not open wild type TRPM3 channel, but when coapplied with PregS, it induces an additional inwardly rectifying component, due to opening of the alternative pore, that is insensitive to La^3+^ block [45]. Unlike the wild type TRPM3 channels, the V990M mutant showed clear opening by clotrimazole alone. In the P1090Q mutant on the other hand clotrimazole did not open the channel, but it reduced currents induced by PregS [36]. In addition, in the V990M mutant, PregS could also open the alternative pore, which manifested as a larger inwardly rectifying current component compared to wild type, which was not blocked by La^3+.^ Opening of the alternative pore thus could also contribute to the higher sensitivity of V990M to PregS [36] and CIM0216 [37]. The alternative permeation pathway is proposed to be located in the S4 segment with potential contributions from S1 and S3 [84]. Thus, the differential effect of the mutations is consistent with their locations, i.e. V990 is in the S4-S5 segment, close to alternative pore region, whereas P1090 is located in S6, far away from S4.

## Gαi coupled receptor regulation of epilepsy-related TRPM3 mutants

Activation of Gαi coupled receptors such as μ-opioid or GABA_B_ receptors inhibits TRPM3 in DRG neurons, an effect mediated by direct binding of Gβγ subunits of heterotrimeric G-proteins [58–60]. Both groups describing the functional effects of the disease-related TRPM3 mutations also tested the effects of Gi-coupled receptor inhibition. Van Hoeymissen et al. co-expressed TRPM3 and its mutant with μ-opioid receptors in HEK293 cells [36]. When the channels were activated with 100 μM PregS, the μ-opioid receptor agonist DAMGO (1 μM) completely inhibited TRPM3 and the P1090Q mutant, while it only caused partial inhibition of the V990M mutant. The concentration of DAMGO required for half-maximum inhibition in the V990M mutant was much higher than that required for the wild type channels, indicating that this mutant is less sensitive to receptor mediated inhibition [36].

Our group obtained similar results on TRPM3 channels co-expressed with M2 muscarinic receptors in Xenopus oocytes. When stimulating channel activity with 50 μM PregS, the P1090Q mutant showed a similar level of inhibition after activating M2 receptors as the wild type group. In contrast V990M was barely inhibited by M2 receptor activation [37]. Considering that the V990M showed dramatically left shifted concentration-responses to PregS, the smaller inhibition could be the result of allosteric effect of the increased sensitivity toward PregS. In order to rule out this possibility, we stimulated wild type and mutant channels using the PregS concentrations corresponding to their EC_50_. Under those conditions, M2 receptor stimulation inhibited the activity of both TRPM3 mutants to the same level as wild type, indicating that the reduced inhibition of V990M was due to allosteric effect of overstimulating the mutant channel with PregS. Taking all the data into consideration, we can conclude that the mutations do not primarily affect Gβγ inhibition, which is consistent with a recent finding that Gβγ binds to a 10 amino acid segment that is far away from both the V990 and the P1090 residues [61] (Figure 1b). Nevertheless, under the same activating conditions, reduced inhibition by Gi-coupled receptor activation may potentially contribute to overactivity of the V990M mutant in neurons.

## Concluding remarks

Two independent publications reported that the two TRPM3 mutations associated with DEE make TRPM3 overactive and likely increase channel activity with distinct mechanisms, which is consistent with the different locations of the affected residues in the channel protein [36,37]. Both papers showed that basal and PregS-induced currents of mutated TRPM3 can be inhibited by the TRPM3 antagonist primidone, which is also a clinically approved antiepileptic [36,37]. The concentrations required to inhibit TRPM3 and its mutants are comparable to the therapeutic plasma levels of primidone [57], suggesting that primidone may be used as a rational therapy for this channelopathy. The data also raise the possibility that the direct inhibition of TRPM3 by primidone contributes to its antiepileptic effects, and that TRPM3 inhibitors may be developed to treat epilepsy in the future.
